# Transcranial brain-wide functional ultrasound and ultrasound localization microscopy in mice using multi-array probes

**DOI:** 10.1038/s41598-025-96647-7

**Published:** 2025-04-08

**Authors:** Mathis Vert, Ge Zhang, Adrien Bertolo, Nathalie Ialy-Radio, Sophie Pezet, Bruno Osmanski, Thomas Deffieux, Mohamed Nouhoum, Mickael Tanter

**Affiliations:** 1 Physics for Medicine Paris, INSERM U1273, ESPCI Paris, PSL University, CNRS, Paris, France; 2 Iconeus, Paris, France

**Keywords:** Functional ultrasound imaging, Ultrasound localization microscopy, Multi-array probe, Cerebral blood volume, Brain imaging, Neuro-vascular interactions, Brain imaging, Ultrasonography

## Abstract

Functional ultrasound imaging (fUS) and ultrasound localization microscopy (ULM) are advanced ultrasound imaging modalities for assessing both functional and anatomical characteristics of the brain. However, the application of these techniques at a whole-brain scale has been limited by technological challenges. While conventional linear acoustic probes provide a narrow 2D field of view and matrix probes lack sufficient sensitivity for 3D transcranial fUS, multi-array probes have been developed to combine high sensitivity to blood flow with fast 3D acquisitions. In this study, we present a novel approach for the combined implementation of transcranial whole-brain fUS and ULM in mice using a motorized multi-array probe. This technique provides high-resolution, non-invasive imaging of neurovascular dynamics across the entire brain. Our findings reveal a significant correlation between absolute cerebral blood volume (ΔCBV) increases and microbubble speed, indicating vessel-level dependency of the evoked response. However, the lack of correlation with relative CBV (rCBV) suggests that fUS cannot distinguish functional responses alterations across different arterial vascular compartments. This methodology holds promise for advancing our understanding of neurovascular coupling and could be applied in brain disease diagnostics and therapeutic monitoring.

## Introduction

The recent advancements in functional ultrasound imaging (fUS) have opened new possibilities for utilizing ultrasound imaging in neuroscience research^1–6^. This technique leverages ultrafast Doppler imaging to estimate cerebral blood flow variations, thereby inferring the associated brain activity through neurovascular coupling^7–9^. Its high resolution, ability to detect responses on a single trial basis, and non-invasive nature make fUS an ideal ultrasound imaging modality for assessing brain function in preclinical animal models such as mice^10^.

Complementarily, ultrasound localization microscopy (ULM) has introduced a super-resolution paradigm in ultrasound imaging, proving its effectiveness in mapping the microvasculature of various organs, including the brain and kidneys^11–13^. By using contrast-enhancing microbubbles (MBs) and ultrafast (several kilohertz) frame rates, ULM circumvents the diffraction-limited resolution barrier, revealing intricate details of hemodynamics even in deeply seated organs^14,15^.

Despite the potential of these imaging modalities, several technical challenges remain, especially the transition from two-dimensional (2D) to three-dimensional (3D) imaging. To expand the field of view, two principle approaches have been proposed: motorized linear 1D probes^16,17^ and 2D matrix probes^18,19^. However, the former lacks the fast-scanning capability necessary for whole-brain imaging at high frame rates, while the latter is often limited in sensitivity in imaging blood flow, particularly for transcranial brain imaging. Bertolo et al. recently introduced a motorized multi-array probe that combines the sensitivity of linear probes with the capability for fast whole-brain scanning^20^. While at early development stage, other promising technologies based on fast moving acoustic mirrors were also recently shown able to transform conventional linear 1D arrays into 3D imaging probes^21^.

Functional ultrasound localization microscopy (fULM) has been established as an advanced tool for high-resolution, functional neurovascular imaging^22^. Previous studies have demonstrated that fULM achieves microscopic spatial resolution on the scale of a few microns across brain-wide regions in murine models, achieving a resolution surpassing that of conventional fUS by more than ten-fold^23^. This enhancement allows for precise visualization of blood flow changes in response to brain activity. Notably, in contrast to conventional ULM, which primarily provides static images of vascular structures, fULM facilitates the dynamic tracking of neurovascular responses at a fine scale. This capability enables the detailed examination of the contributions of distinct vascular compartments, including arterioles, capillaries, and venules, during task-evoked brain activation. However, several practical limitations of fULM remain. First, fULM does not offer the functional connectivity mapping demonstrated previously with fUS, limiting its application in studying brain-wide functional interactions. Second, fULM typically requires a significantly longer acquisition time compared to conventional ULM, posing challenges for real-time or large-scale applications. Integrating fUS and ULM within a multi-array probe offers a promising approach to address these limitations. Such a configuration could potentially enable both the mapping of functional connectivity and a reduction in acquisition time, thereby expanding the scope and efficiency of neurovascular imaging beyond the current capabilities of fULM.

Over the past decade, the concept of neurovascular coupling has significantly evolved to account for the cellular and structural diversity across various vascular compartments within the cellular vascular tree^24^. Literature suggests that the blood recruitment chronology goes from the smallest arterial compartments -capillaries and parenchymal arterioles- to the biggest -pial arterioles and cerebral arteries. While fUS can effectively estimate brain activity through neurovascular coupling, it remains unclear whether fUS alone could differentiate responses across different arterial levels, from pial arterioles and cerebral arteries to capillaries and parenchymal arterioles, for comprehensive functional analysis as its spatial resolution is typically limited to 100 μm and may not allow to separate these different vascular compartments. Consequently, it is interesting to perform fUS and ULM on the same imaging target to investigate whether functional activation in response to whisker stimulation correlates with changes in cerebral blood volume measured by fUS or with microbubble speed estimated through ULM. The motorized multi-array probe combines the high sensitivity of linear probes with fast scanning capabilities for whole-brain imaging, making it ideally suited for this study.

The proposition of a single ultrasonic probe able to perform both 3D fUS imaging and 3D ULM imaging on the same animal without compromising their individual performances is important for neuroscience studies. In this study, we built upon these former works by demonstrating the feasibility of using such a probe to perform both fUS and ULM transcranially, enabling non-invasive whole-brain functional imaging in mice in conjunction with microvascular imaging and hemodynamic quantification at microscopic scale. We introduce an improved motorized scheme that optimizes imaging ratio (89% imaging ratio, with 10 s of imaging followed by 1.2 s of movement), facilitating enhanced MB detection within their relatively short lifetime. Additionally, we demonstrate that the resulting ULM maps encompass the whole-brain field-of-view, as achieved with fUS (11.9 × 7.87 × 8.1 mm^3^). We further investigate the registration of functional and anatomic data, allowing for detailed analysis of vascular structures in task-specific functional areas.

## Methods

### Ethics

All procedures were performed on six male C57BL/6 J mice (7 weeks old, Janvier labs, France in compliance with European regulations and approved by the local ethics committee (Comité d’éthique en matière d’expérimentation animale number 59, Paris Centre et Sud, project number.

25358-2020051019027581V2). The mice were housed three per cage with a 12-h light/dark cycle, a constant temperature of 22°℃ and a humility level between 45 and 50%. Unlimited access to food and water was provided. Before the experiments, animals were given a 1-week minimum acclimatization period to housing conditions. All experiments followed ARRIVE guidelines and relevant animal care regulations.

### Animal preparation

Anesthesia was induced with 2% isoflurane (delivered via a nose mask, Minerve, France) and maintained using intraperitoneal medetomidine injection (80 µg/kg). The fur on the mice’s heads was shaved to prevent air bubbles within the ultrasound gel. Mice were fixed in a stereotaxic frame, and their eyes were protected with moisturizing gel (Ocry-Gel, Virbac, France). Body temperature was maintained at 37°℃ using a heating bed, monitored by a rectal probe (Physitemp, Clifton, USA). Heart rate and respiratory rate were monitored using a PowerLab data acquisition system with LabChart software (ADInstruments, USA). An intramuscular injection of medetomidine (100 µg/kg) was performed after catheterization of the saphenous vein for microbubble injections (Sonovue, Bracco, Italy).Anesthesia levels were carefully adjusted to maintain stable physiological parameters, with isoflurane levels ranging from 0 to 0.5% and continuous medetomidine infusion at 100 µg/kg/h.

### Multi-array probe

**Fig. 1:**
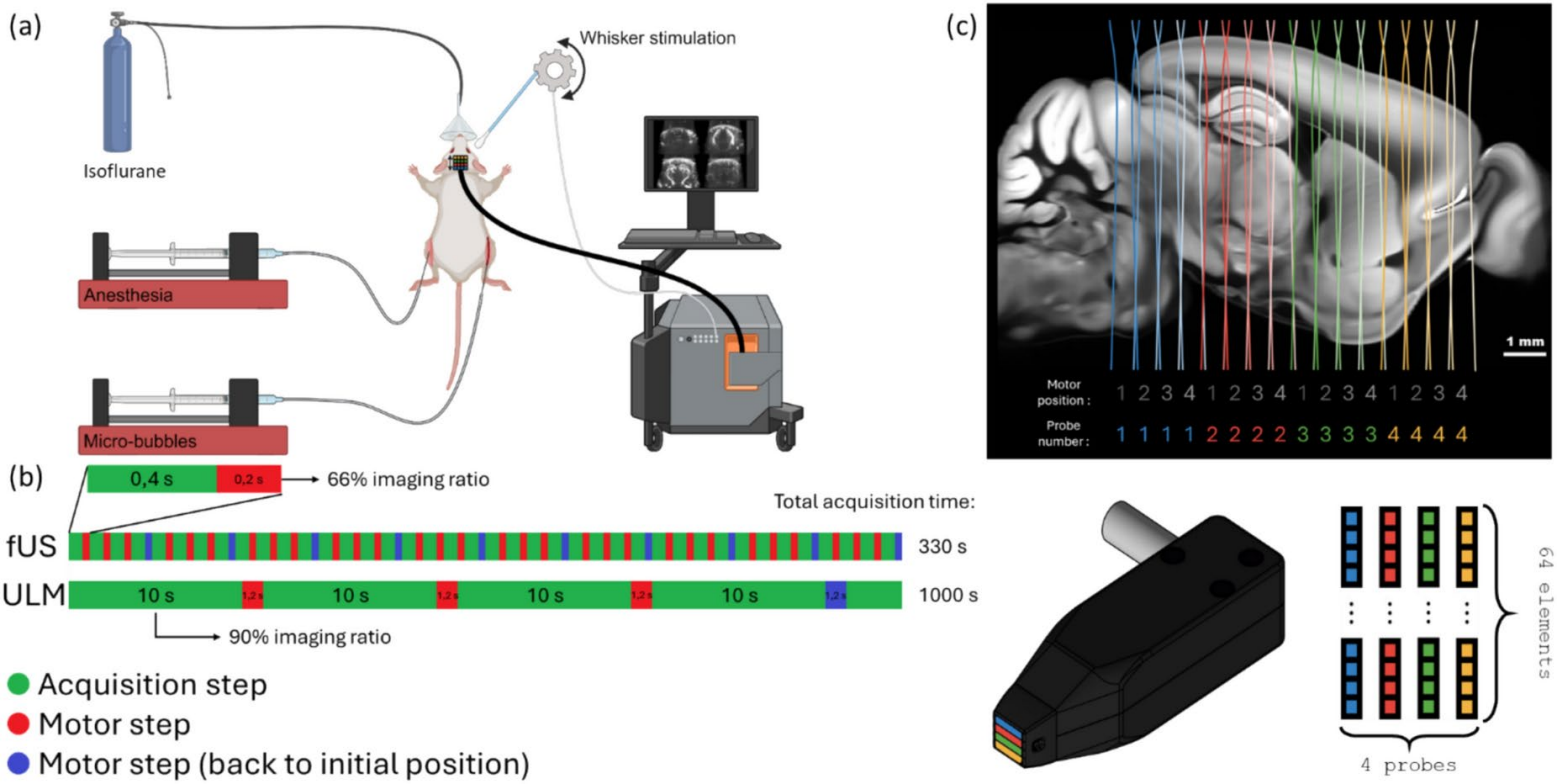
(**a**) Setup schematic. Images were acquired using a preclinical ultrasound scanner. (**b**) The proposed motor pattern enables 89% acquisition ratio to optimize the microbubble detection. (**c**) The combination of a multi-array probe and a motorized setup enables fast whole brain scanning. The multi-array probe is composed of 4 linear 64 elements sub probes (15 MHz).

Data was acquired transcranially using a multi-array ultrasound probe composed of four 64 elements linear sub-arrays with a center frequency of 15 MHz and a 0.11 mm pitch (IcoPrime-4D Multi-array, Iconeus, Paris, France). This probe was specifically designed for whole-brain fUS^20^. The probe was driven by a preclinical ultrasound system (Iconeus One, Iconeus, Paris, France) and mounted on a 4-axis motor system for rapid whole-brain scanning. The motorized setup moved in 525 µm steps—equivalent to one-quarter of the sub-array distance—enabling coverage of the whole brain with each step, as illustrated in Fig. 1c. The imaging depth was approximately 8 mm, with a slice thickness of 500 µm. The high sensitivity of this probe is attributed to several factors, including the large active surface area of each element (1.5 mm width), the small pitch (0.11 mm), and the acoustic lens beneath each array, which focuses acoustic energy on a slice approximately 500 microns thick (minimum thickness at an 8 mm depth), similar to linear arrays. The four independent linear arrays are tightly assembled with a 2.1 mm spacing between each array, minimizing acoustic cross-talk and optimizing the field of view.

The multi-array probe was mounted on a 4-axis motorized stage to allow precise positioning and scanning. For the purposes of this study, the translation of the probe was restricted to a purely linear motion in a single dimension, enabling the sequential acquisition of imaging planes. The motorized stage was programmed to move the probe in discrete steps with a high degree of accuracy, ensuring consistent coverage of the entire brain volume while maintaining alignment between the acquired planes.

### Imaging acquisition and beamforming

Functional data was captured using the scanner’s live acquisition software (IcoScan, Iconeus, Paris, France). ULM data was acquired using a custom version of the software. For both fUS and ULM acquisitions, a mechanical index of 0.1 was used to prevent microbubble destruction during ULM while maintaining adequate sensitivity for fUS to counteract signal attenuation caused by the skull. All data was acquired using tilted plane-wave imaging sequence with 10 angles compounded at 500 Hz. Beamforming was performed on a trapezoidal shape to enlarge the field of view using the maximum tilt angle of ± 12°. The experimental setup can be seen from Fig. 1a.

The imaging sequence was implemented using ultrafast plane wave transmission (Transmit frequency: 15 MHz, Pulse widths: 2 cycles per pulse) and reception schemes replicated for each of the four linear arrays. Each subarray (4 × 64 elements) was tightly spaced with a 2.1 mm distance between subarrays to optimize the field of view while minimizing cross-talk. Four images were simultaneously obtained from the corresponding four linear arrays at 500 Hz using 10 tilted plane waves acquired at a PRF of 4 kHz.

The total acquisition times, including the probe’s linear movements, were 330 s for fUS and 1000 s for ULM, as illustrated in Fig. 1b. During each session, the motorized stage ensured precise and consistent probe positioning, with steps matched to one-quarter of the subarray spacing, as shown in Fig. 1c. This approach enabled coverage of the whole brain field of view (11.9 × 7.87 × 8.1 mm^3^).

### Whisker stimulation and evoked response acquisitions

For fUS acquisitions, a mechanical index of 0.1 was used to enhance sensitivity and counteract signal attenuation due to the skull. The motor was programmed for 0.4-s acquisition periods, followed by 0.2-s movement intervals, resulting in a volume acquisition time of 2.4 s (0.42 Hz volume rate). Data from the four sub-arrays were concatenated for whole-brain analysis. During fUS acquisitions, mice whiskers were stimulated at 3 Hz using a cotton bud mounted on a servomotor controlled by an Arduino board, synchronized with the ultrasound scanner. Two stimulation protocols were used: 30 s baseline, 30 s ON, 30 s OFF (repeated five times), and 20 s baseline, 10 s ON, 20 s OFF (repeated ten times). Data from two animals subjected to the 10 s ON / 20 s OFF protocol were excluded due to artifacts. The excluded data were affected by imaging artifacts, which were evident in the fUS images. These artifacts may have been caused by several factors. First, non-uniform application of the coupling gel during scanning could have led to uneven acoustic transmission, resulting in inaccurate estimation of functional activation areas. Second, variations in skull thickness or the presence of sutures could have contributed to these artifacts due to inconsistent acoustic reflection.

### Ultrasound localization microscopy processing

After completing fUS acquisitions, ULM imaging was performed to achieve microscopic-resolution angiography, conducted in the absence of whisker stimulation. Sonovue© microbubble contrast agents (Bracco, Italy) were injected at a rate of 1.5 mL/h through the great saphenous vein using a syringe pump (KDS Legato 130, KD Scientific, Holliston, USA) as described in the previous literature^25^. To optimize acquisition time, a customized motor protocol (Fig. 1b) was implemented, achieving a 89% imaging ratio (10 s acquisition, 1.2 s motor movement). Data was collected over 1000 s, yielding 220 s of cumulative imaging per plane. Microbubble tracks were processed through a filtering-localization-tracking pipeline, achieving an in-plane resolution of 5 × 5 µm^11,26^.

Fourier ring correlation analysis was applied on ULM image to quantify the resolution limit of the final ULM image^27^. Briefly, all of the localization events were split into two subsets, and thus two sub-ULM images. The correlation of the spatial frequency content is then calculated as the normalized correlation of the two spectrum along iso-spatial frequency ring.

### General linear model for whisker stimulation

To detect activated brain areas during whisker stimulation, we compared the fUS signal in each voxel with the stimulus pattern. For this purpose, we computed z-scores based on general linear model analyses (GLM) with Bonferroni correction for multiple comparisons. The GLM approach is the gold standard analysis approach in functional MRI studies and was used here in an effort to standardize results across modalities^28^. Power Doppler signals were adjusted to eliminate baseline activity and enable the quantitative estimation of relative CBV (rCBV). The baseline was estimated using the average value of the power Doppler signal on a per-measurement basis to ensure consistency and accuracy in the analysis. Expected stimulus responses were modeled by convolving the stimulation pattern with a modeled impulse response of the cerebral flow following stimulation, as described in^29^. The design matrix used to detect cortical activation specific to the whisker stimulation thus included the stimulus pattern as a regression signal and a constant slope to model signal drift. Statistical parametric maps were generated for each session, including a z-map, a p-value map, a stimulation map, and a baseline map. Individual delta cerebral blood volume (ΔCBV) maps were obtained by dividing the stimulation matrix by the baseline matrix. To determine which pixels achieved statistical significance considering the comparison of all the pixels in the z-map, we considered p-value < 0.05 statistically significant and further performed Bonferroni correction for multiple tests comparison. The pixels meeting the defined criteria were considered activated. Statistical significance was determined using a t-test, and results were converted to z-scores for inter-subject comparison. P-values were corrected using the Bonferroni method.

### Data registration on brain functional atlas

A brain position system (BPS) was leveraged to align the acquisitions to the Allen atlas before quantification^30^. Basically, a 3D Power Doppler volume was acquired at the beginning of each imaging session and then registered to a reference vascular map previously aligned with the brain atlas. This enables subsequent fUS acquisition of the same imaging session to be automatically aligned with the brain atlas for brain regions segmentation. As the fUS and ULM acquisitions were performed consecutively to be aligned during the image session, the same registration process was used for ULM acquisitions. However, as the BPS system was performed using Power Doppler, the registration process average accuracy was approximately 100 μm. Thus, we performed additional manual registration to improve ULM acquisition alignment to the atlas.

### Group-level statistics

For each fUS acquisition (n = 10), quantitative rCBV and associated t-statistic were correlated with underlying MB speed. The obtained correlation coefficients were adjusted using the Fisher transform to enable group averaging. The Fisher transform converts correlation coefficients, which are bounded between -1 and 1, into values that are approximately normally distributed. This transformation allows for more accurate statistical analysis and comparison across groups by stabilizing variance and ensuring the validity of parametric statistical tests. The metrics (rCBV and t-statistic) were tested against the hypothesis of a zero-mean using a Wilcoxon signed-rank test. The Wilcoxon signed-rank test is a non-parametric test that does not assume a normal distribution of data. Therefore, it is suitable for providing robustness in assessing central tendencies of small sample sizes without strict distributional assumptions. Additionally, as it is a paired test, it focuses on the differences between paired observations, thus making it a sensitive test for detecting differences between two pairs of samples.

## Results

### Multi-array probe enables whole-brain microscopic angiography with ULM

**Fig. 2:**
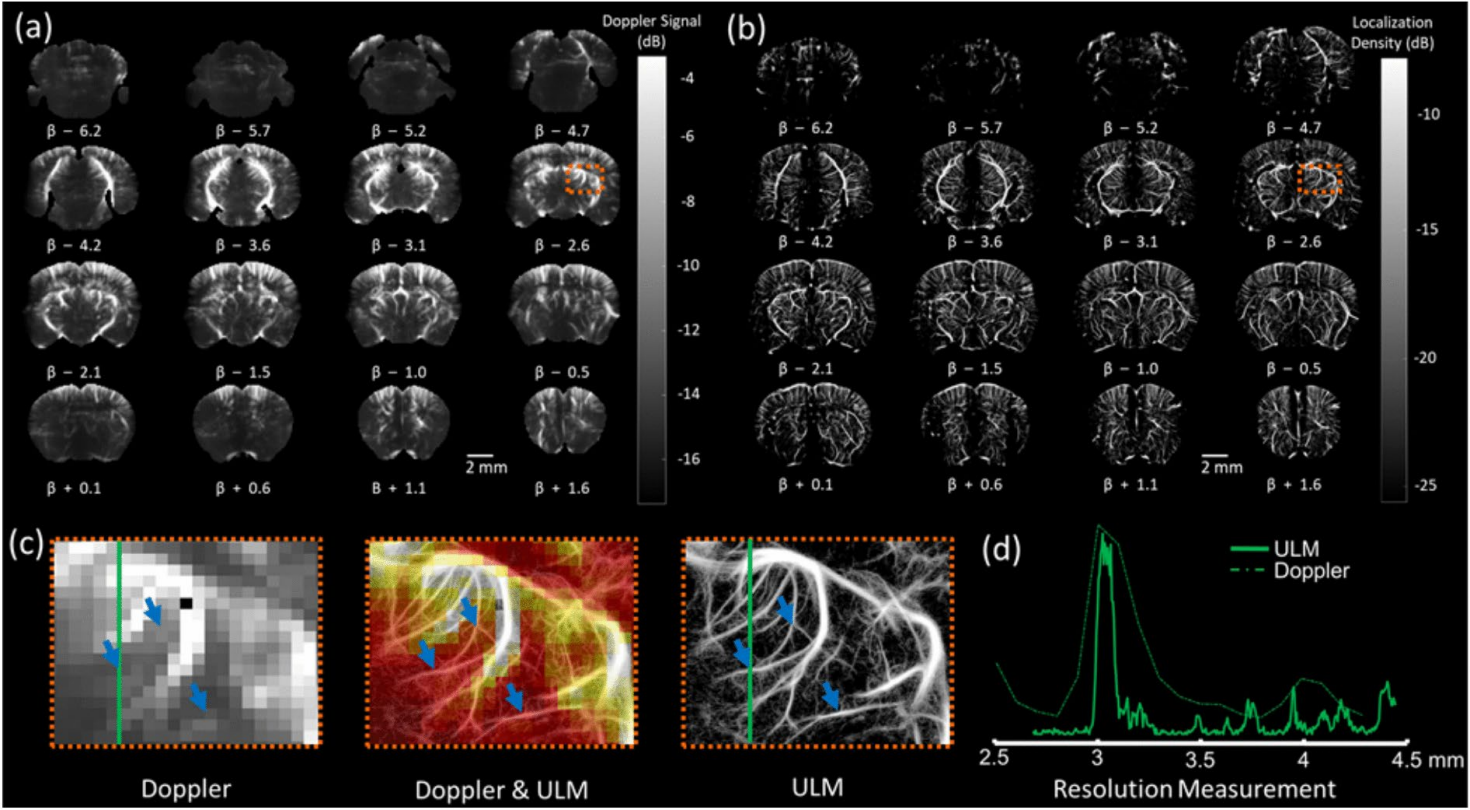
Comparison between Doppler image and ULM localization maps, covering a field of view of 11.9 × 7.87 × 8.1 mm^3^ across 16 imaging planes. β represents the z-position of each imaging plane with respect to bregma. (**a**) Time-averaged Doppler images highlight major vascular structures at a resolution of 110 × 98 × 525 µm. The colorbar represents the measured Doppler signal in dB. (**b**) Microbubble localization maps provide a more detailed representation of microvascular features at 5 × 5 × 525 µm resolution. The colorbar represents the measured MB localization signals in dB. (**c**) Overlaid Doppler and ULM images acquired on the same imaging plane demonstrate how ULM captures finer microvascular structures, as indicated by blue arrows. These arrows mark regions where ULM reveals microvasculature which cannot be visualized in Doppler images. (**d**) The intensity profile, drawn along a green line, contrasts the resolution limits of Doppler and ULM, with Doppler resolving vessels down to 234 µm and ULM detecting structures as small as 32 µm.

The time-averaged Doppler signal from fUS and the rasterized MB counts from ULM acquired on the same imaging plane are shown in Fig. 2a,b for comparison. These images, acquired in two different modes, illustrate how the multi-array probe can scan the whole brain across 16 imaging planes. Both imaging modalities were performed during a single acquisition session using the same setup (probe, motors, scanner, and anesthesia), allowing the assessment of brain function using fUS and detailed microvascular anatomy using ULM. Doppler images demonstrate high sensitivity to blood flow, except in the first three frontal slices where the skull absorption and aberrations impede transcranial imaging. Doppler sensitivity plays a critical role in functional analysis by inferring neuronal activation based on variations in CBV signal over time. The Doppler images result from averaging signals over a task-based acquisition lasting 330 s, as can be seen from Fig. 1b. ULM localization map enables high-resolution mapping of microvasculature, resolving vessels as small as 32 µm, as can be seen from Fig. 2d. They are the result of the rasterization of 2.5 million bubbles tracked in 1000 s. The local comparison of Doppler and ULM maps in Fig. 2c shows how ULM reveals vessels hidden in Doppler maps, much smaller than the pixel size. In Fig. 2c the blue arrows point to regions where the vessels do not raise contrast in Doppler images while being well defined in ULM. The intensity profile along the green line was extracted from both maps and compared in Fig. 2d.

### Whole-brain quantification of blood flow properties via MB tracking

**Fig. 3:**
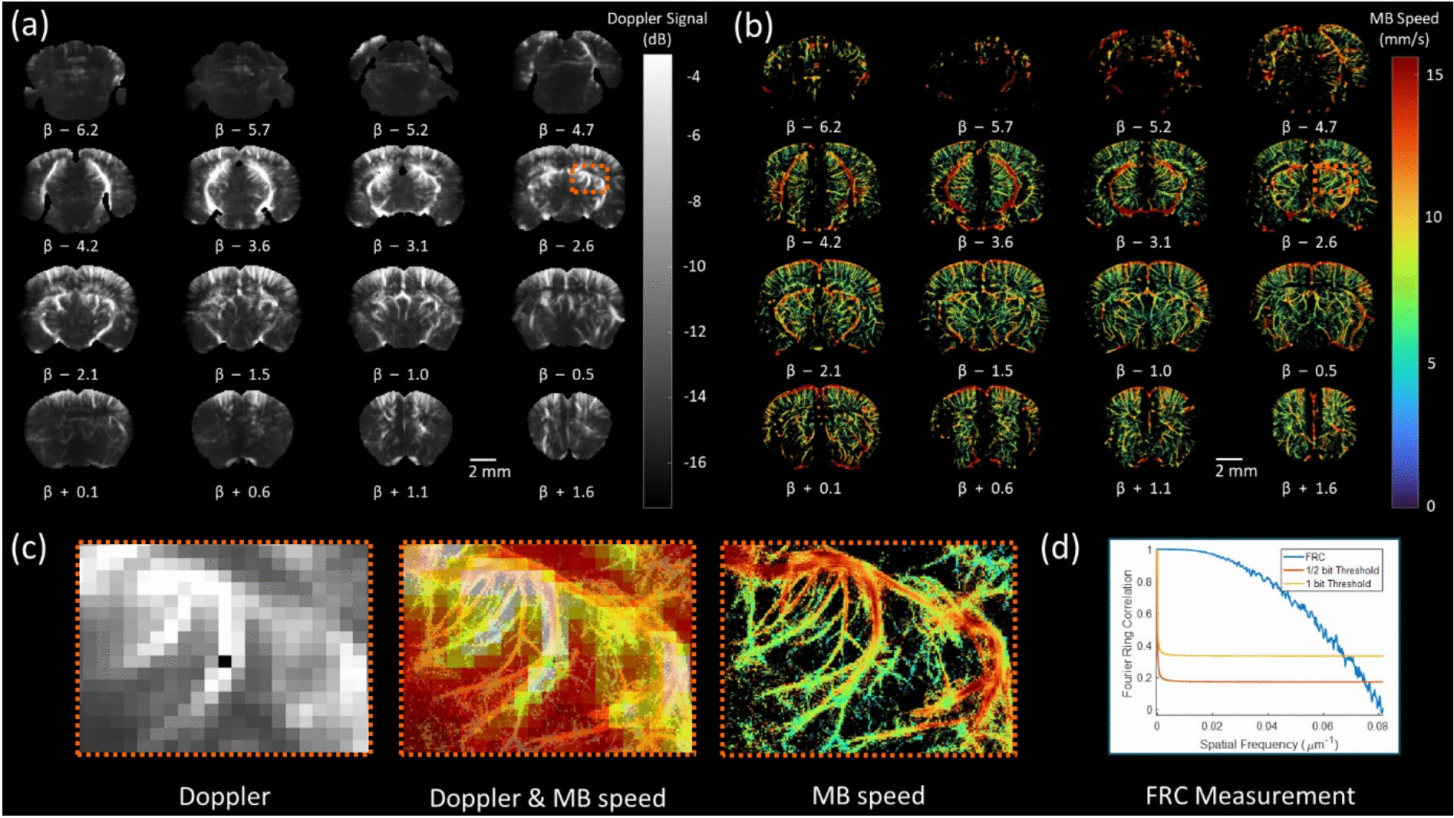
Comparison between Doppler image and ULM speed maps, covering a field of view of 11.9 × 7.87 × 8.1 mm3 across 16 imaging planes. β represents the z-position of each imaging plane with respect to bregma. (**a**) Time-averaged Doppler images highlight major vascular structures at a resolution of 110 × 98 × 525 µm. The colorbar represents the measured Doppler signal in dB. (**b**) Microbubble speed maps provide a more detailed representation of microvascular features and blood flow speed at 5 × 5 × 525 µm resolution. The colorbar represents the measure MB speed in mm/s. (**c**) The combined Doppler and ULM images acquired on the same imaging plane reveal additional vascular structures captured by ULM. (**d**) Fourier ring correlation analysis on ULM image, demonstrates a resolution limit of 13.33 µm.

In Fig. 3a, time-averaged Doppler images effectively depict major vascular structures, achieving a spatial resolution of 110 × 98 × 525 µm. This resolution captures primary vasculature but lacks the details needed to resolve finer microvascular networks. By contrast, Fig. 3b showcases microbubble speed maps obtained through ULM, revealing a highly detailed representation of microvascular flow information with a significantly enhanced resolution of 5 × 5 × 525 µm. This improvement enables visualization of microvascular flow information that Doppler imaging cannot distinguish. In Fig. 3c, the combined Doppler and ULM images provide complementary insights, highlighting additional vascular structures detected by ULM, thus enhancing the depth of structural detail across the vascular network. Lastly, Fig. 3d employs Fourier ring correlation analysis on the ULM image, determining a resolution limit of 13.33 µm. This analysis substantiates the superior resolving power of ULM for microvascular imaging, achieving a level of detail that Doppler imaging alone cannot provide.

### ULM reveals microvasculature in functionally active regions

**Fig. 4:**
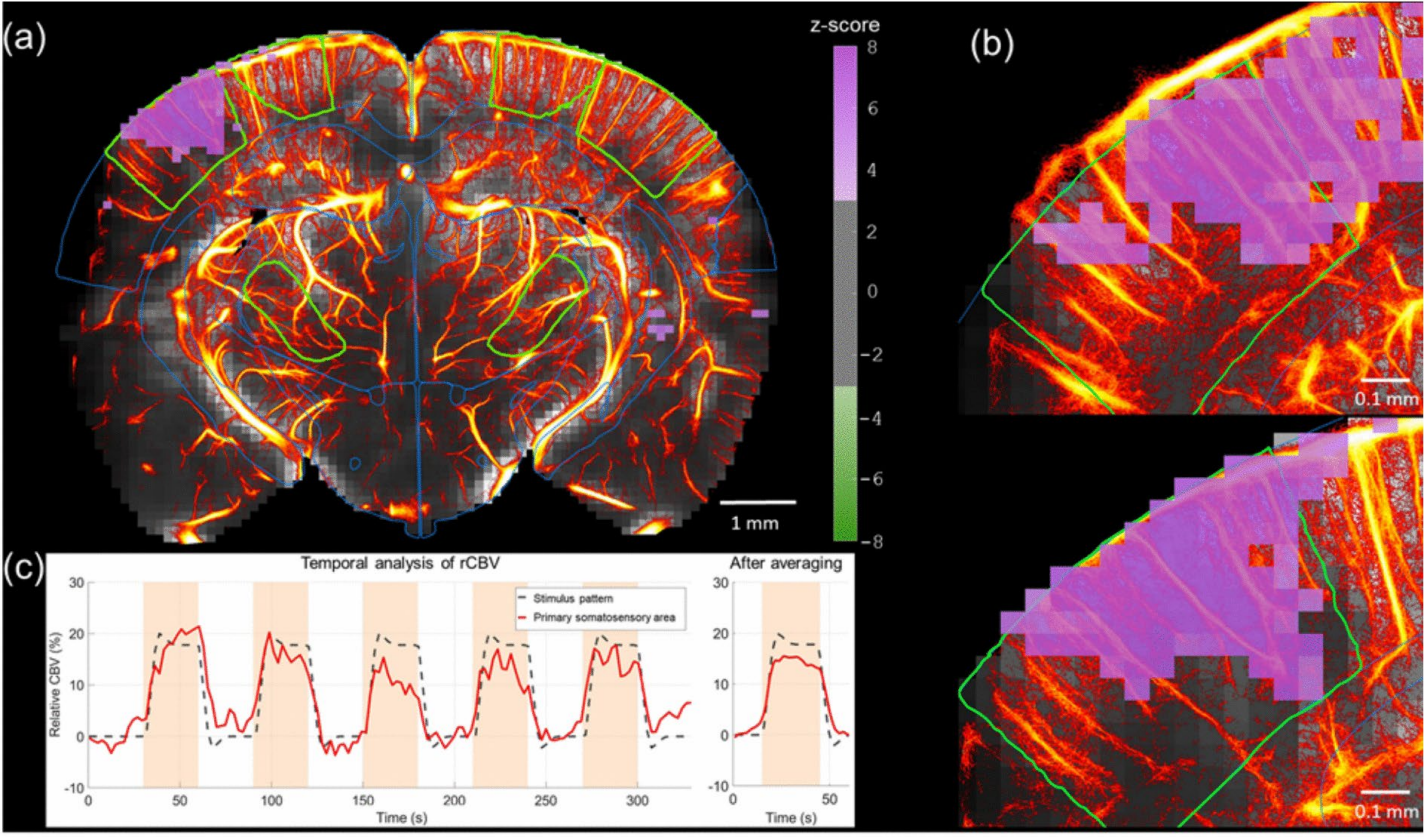
Demonstration of ULM exposes the microvasculature underlying functional brain regions, in response to whisker stimulation. (30-s ON, 30-s OFF). (**a**) A brain slice intersecting the barrel cortex, with a z-score map highlighting the contralateral cortical response to whisker stimulation. (**b**) Zoom-in images of functionally active areas from two animals demonstrate the reproducibility of the stimulation and its mapping to the expected region. (**c**) Temporal analysis shows strong correlation between the barrel cortex signal and the stimulation pattern, with a 15% relative increase in CBV during whisker stimulation. The temporal analysis after averaging can be seen on the right.

Figure 4 summarizes the results of the assessment of the brain function through whisker stimulation and the underlying network of micro vessels. The maps shown in purple display the z-score of the pixel-wise correlation between the task stimulus and the measured Doppler signal. The measured response to the stimuli is significant for single trial fUS acquisition. The significantly activated area fits the expected functional region, i.e. the barrel field region of the cortex (as can be seen from Fig. 4a,b). Experiments were repeated on 10 animals and were found to be reproducible (One-way ANOVA analysis of rCBV suggests a p-value of 0.17 among n = 10 animals). Also, the spatially averaged time signal of the barrel field is highly correlated with the temporal track of the stimulus (Fig. 4c). The measured relative increase of CBV in this region reaches 15% during the stimulation.

### Group-level statistics reveal the hierarchy of functional responses across vessels

**Fig. 5:**
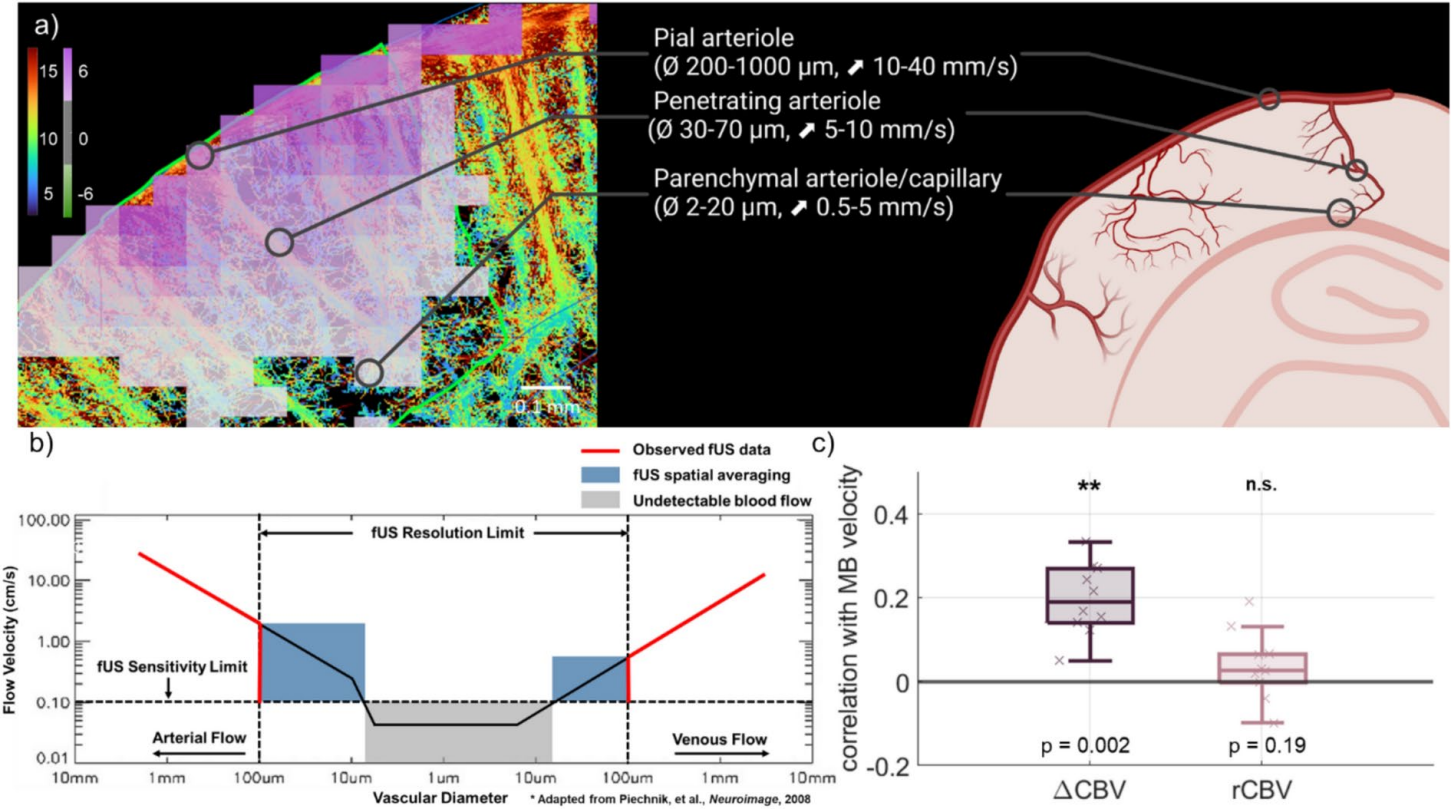
Relationship between vessels rank and functional response, using MB speed as a proxy for vessel hierarchy. (**a**) Overlay of the relative CBV increase in the barrel field and the MB speed, delineating different arterial compartments based on diameter and speed. (**b**) fUS sensitivity and resolution thresholds draw the limits of detection of the vascular tree based on its spatial features (flow speed and diameter dependence corresponds to (b) from^31^). The solid black line defines the slowest flows that fUS is sensitive to. Dotted black line defines the voxel size in which any sources of signal are averaged (green area). (**c**) Distribution of the correlation of ΔCBV and rCBV with MB speed. The absolute evoked response (ΔCBV) is correlated with measured speed. The response significance (rCBV) is decorrelated from the flow due to the lack of fUS resolution on the lowest arterial branches. (*: p < 0.05, **: p < 0.01, n = 10 acquisitions, 6 mice).

The neurovascular response to a stimulus is supposedly varying depending on the arterial level. As shown in Fig. 5a, ULM speed maps can describe such a hierarchy of arterial vessels while Doppler and thus fUS images do not. However, while fUS does not have the spatial resolution to distinguish this range of vessels, it still is sensitive to slow flows down to 1 mm/s (Fig. 5b)^31^. We can interpret this as a spatial averaging of the signal coming from arterioles level 1 to 4 (see green rectangle) in 100 × 100 × 525µm^3^ voxels. As demonstrated in Fig. 5c, over the cohort (n = 10), the absolute increase of CBV (ΔCBV) is significantly weakly correlated with the MB speed (r = 0.19, p = 1.9 × 10^−3^). This means that the strength of the evoked response is correlated with the vessels level. On contrary, the relative response—measured as the relative increase of CBV- is on average not correlated with the speed.

## Discussion

This study demonstrated the successful application of functional ultrasound imaging (fUS) and ultrasound localization microscopy (ULM) using a motorized multi-array probe to achieve transcranial whole-brain functional and microvascular imaging in mice. By combining these two modalities in a single setup taking benefit of the high sensibility of the multi-array probe technology, it was able to capture both functional and anatomical data and introduce a tool that may offer comprehensive insights into the cerebral microvasculature and its role in neurovascular coupling. The optimized motorized scanning scheme allowed for a 89% imaging ratio, enhancing microbubble (MB) detection, while the ULM maps provided detailed microvascular structures not visible in power Doppler images. Our results indicate that functional activation in response to whisker stimulation was strongly correlated with changes in cerebral blood volume (CBV).

Former works in fUS imaging suggested that including sub-spectrum Power Doppler separation in fUS data analysis could provide a spatial segregation of the brain hemodynamic activity between larger vessels and capillaries as higher frequencies in the Doppler signal describe large speed in large arterioles and lower frequencies would describe slower speed in capillaries^32,33^. Such sub-spectrum analysis applied to the fUS data would thus offer a way to separate the rCBV response of capillaries from the rCBV response of larger vessels. As it was already shown that the rCBV response in first order capillaries^23,34^ is the major contributor to the neurovascular response, we should here find that the rCBV response obtained in fUS imaging is higher in activated regions with low MB speeds and small capillaries. This is not what is found by comparing rCBV assessed with fUS with MB speed microscopic maps obtained on the same animals using ULM (Fig. 5a,c). We rather show that the relative CBV increase is not correlated with MB speed. This suggests that Doppler-based filtering of low velocities based on sub-spectrum Power Doppler filtering is not be sufficient to extract the complexity of the neurovascular response in particular the discrepancy of relative neurovascular response in arterioles and smaller capillary vessels. The limited 100 µm resolution of fUS imaging is to date not sufficient to separate each vascular compartment. In particular the slow flow near the arteriole vessel walls can simply not be separated from slow flow in capillaries (Fig. 5a).

As can be seen from Fig. 5, the vessels hierarchy from ULM maps and the observed spatial distribution of vessels and functional response (shown in red line) can be inferred given by fUS. In other words, if we make the strong assumption that small velocities correspond to small vessels, it could potentially be possible to distinguish the functional response of small vessels from the one of larger arterioles by separating these components in the Doppler Fourier spectrum. To evaluate if such inference was possible from the data, we measured the correlation between the fUS functional response and speed of the underlying microvessels blood flow. We considered the MB speed as a proxy of the speed of blood flow and consequently of the vessel hierarchical level. The results demonstrated in Fig. 5c suggest that this result denies the possibility to distinguish the response of different arterial levels in the Doppler data. Indeed, both two-photon optical imaging and functional ULM have shown that the relative CBV increase during stimulation should be much higher in the Arteriolar-Capillary Transition (ACT) zone than in the penetrating arterioles^22,35–37^. We clearly see in Fig. 5a that the rCBV change is not higher in the regions located in between two penetrating arterioles compared to the rCBV change in pixels located in a penetrating arteriole.

Neurovascular coupling, the process by which neuronal activity is linked to changes in cerebral blood flow, requires detailed knowledge of both vascular structure and flow dynamics. Conventional techniques such as fMRI provide only macroscopic views of this process, whereas fUS allows for high-resolution, real-time measurement of blood flow changes in response to neuronal activation^38^. ULM, with its ability to track microbubbles and reveal capillary-level details, adds a new dimension by mapping the microvascular network responsible for these changes. By correlating functional activation data (from fUS) with microvascular structure and MB speed (from ULM) across 16 different imaging planes obtained using multi-array probe, this study enhances our understanding of how different vascular compartments contribute to neurovascular coupling. Furthermore, the ability to distinguish between different vessel sizes and flow rates also reveals how blood is recruited during neuronal activation. The ability to simultaneously acquire high-resolution data across multiple imaging planes allows us to rapidly distinguish between vessel sizes and flow dynamics across different regions, offering novel insights into how blood flow is modulated at both macro- and microvascular levels. This unified approach underscores the complementary roles of fUS, ULM, and the multi-array probe in advancing our understanding of brain function.

The non-invasive nature of fUS and ULM, combined with their ability to provide high-resolution microvascular maps, makes them ideal tools for longitudinal studies. These techniques allow for repeated imaging of the same animal over time, enabling researchers to monitor changes in the microvasculature under different conditions. This could be particularly useful for studying microvascular alterations in neurodegenerative diseases, stroke recovery, or chronic conditions such as diabetes^39^. Monitoring changes in vessel density, and vascular dynamics over time would provide valuable insights into disease progression and the effects of therapeutic interventions. Longitudinal studies using fUS and ULM could also be applied to track vascular plasticity in response to environmental or behavioral changes, further deepening our understanding of brain health and resilience.

The ability to differentiate between various vascular and neuronal pathologies is one of the most promising clinical applications of fUS and ULM. Vascular dementia, for example, is primarily caused by reduced blood flow to the brain, often due to microvascular damage^40^. ULM’s ability to map microvascular structures with high resolution could be instrumental in distinguishing these conditions. By quantifying changes in microvascular density and flow patterns, ULM and fUS could help identify specific vascular abnormalities that are characteristic of vascular dementia, such as microinfarcts or capillary loss. This would allow for earlier and more accurate diagnosis, enabling better-targeted interventions for each condition^41^.

Vascular plasticity refers to the brain’s ability to remodel its vascular network in response to changes in neuronal activity, metabolic demand, or injury. This plasticity is critical for maintaining proper brain function, particularly in aging and disease^42^. The ability of ULM to visualize and quantify microvascular dynamics at high resolution provides a unique opportunity to study this process. By tracking changes in vessel diameter, flow dynamics, and microvascular density over time, ULM can reveal how the brain adapts to new demands or compensates for damaged regions. Furthermore, longitudinal studies using fUS and ULM could investigate how vascular plasticity is affected by various factors, such as aging, exercise, or pharmacological treatments, providing new insights into therapeutic strategies for maintaining brain health^43^.

The multi-array probe comprises four compact linear arrays, each with 64 elements, enabling simultaneous acquisition of four images corresponding to the four arrays. This configuration improves time resolution by a factor of four while maintaining sensitivity and spatial resolution. Additionally, comparative imaging quality between the multi-array probe, row-column addressed (RCA) array probe, and fully populated matrix (FPM) array probe using the same animal model has been explored in previous studies. The findings indicate that, compared to RCA and FPM probes, the multi-array probe consistently provides superior contrast-to-noise ratios and enhanced vessel intensity profile contrasts under both trepanned and transcranial conditions. These characteristics underscore the utility of the multi-array probe in accelerating volumetric imaging without compromising image quality, making it particularly advantageous for fUS and ULM applications.

A number of limitations must be considered when interpreting the results. First, certain regions of the brain remain challenging to image due to stronger skull aberrations, primarily caused by skull shape or the presence of sutures. Implementing aberration correction techniques may help mitigate these issues and improve signal recovery. Second, the current field of view, optimized specifically for the adult mouse brain, would be insufficient for imaging larger animals such as rats. Future advancements, including the development of 512-channel scanners with greater computational power, could facilitate the creation of larger multi-array transducers to address this limitation.

This study demonstrates the feasibility of performing whole-brain fUS and ULM using a motorized multi-array probe in mice. By integrating these advanced imaging techniques, we captured both functional and anatomical data, providing a comprehensive view of neurovascular dynamics across the mouse brain. The motorized scanning scheme enabled a high imaging rate, optimizing microbubble detection and producing high-resolution ULM maps that revealed intricate microvascular details beyond the resolution of Doppler images. Additionally, our findings suggest that rCBV changes, observed in response to stimuli, were not accompanied by significant speed-based differentiation across vascular compartments. This observation aligns with previous reports, indicating that fUS alone may have limitations in distinguishing vascular compartments^23^. This also reinforces the need for combined imaging techniques to fully capture the complexity of neurovascular coupling. This integrated approach enhances our understanding of neurovascular coupling by revealing the hierarchical recruitment of blood flow across different vascular compartments. It holds promise for future applications in both preclinical and clinical neuroscience. The methodology could be applied to longitudinal studies monitoring microvascular changes in neurodegenerative diseases or assessing vascular plasticity. The ability to differentiate functional vascular changes may also aid in diagnosing cerebrovascular pathologies, such as vascular dementia and neurodegenerative diseases altering the neurovascular coupling, such as Alzheimer’s disease, thus advancing the potential of this imaging modality for clinical translation.

## Data Availability

Data supporting the findings of this study are available in the framework of an official collaboration between academic institutions. Correspondence and requests for data should be addressed to M.T.
